# Depression Among First- and Fifth-Year Medical Students in Riyadh, Saudi Arabia

**DOI:** 10.31661/gmj.v8i0.1497

**Published:** 2019-06-10

**Authors:** Abdulaziz Almalki, Abdullah Almalki, Abdullah Kokandi, Bandar Aldosari, Abdulaziz Bin Baz, Shoog Alfadhel, Abdulaziz Alsuwayyigh, Rgad Alsadoun, Bandar Haddad

**Affiliations:** ^1^College of Medicine, Imam Muhammed Ibn Saud Islamic University, Riyadh, Saudi Arabia; ^2^College of Medicine, King Saud bin Abdulaziz University for Health Sciences, Riyadh, Saudi Arabia; ^3^Clinical Neuroscience Department, College of Medicine, Imam Mohammed Ibn Saud Islamic University, Riyadh, Saudi Arabia

**Keywords:** Depression, Medical Students, Cross-Sectional, Beck’s Depression Inventory Questionnaire, Saudi Arabia

## Abstract

**Background:**

Depression, which is characterized by persistent sadness, is a highly prevalent and serious medical disease that affects more than 300 million individuals worldwide. It is hypothesized that the onset of depressive symptoms in medical students occurs due to prolonged exposure to the stressful environment of medical colleges. Hence, we aimed to determine the presence of depressive symptoms and compare the level of depression between the first-year and fifth-year medical students in Riyadh city, Saudi Arabia.

**Materials and Methods:**

This cross-sectional study was performed with convenient sampling from 2 medical colleges in Riyadh in the Kingdom of Saudi Arabia from October to November 2017. The English version of Beck’s Depression Inventory questionnaire was used in this study. Statistical analysis was administered using SPSS via chi-square test, and P-value less than 0.05 was considered statistically significant.

**Results:**

The questionnaire was answered by 241 participants. The age variable shows a bimodal distribution. The mean age of first-year and fifth-year students was 19±0.8 years and 23±2.1 years, respectively. Fifth-year students comprised 53.5% of the total sample, and male students accounted for 63.1% of the total sample. After analysis, the results show that male students scored higher than female students on the lower side of the scale in both years. Most of the first-year female students scored a higher level of depression compared with male students (P=0.001), whereas no difference between males and females (P=0.04) was found in the fifth-year students’ data.

**Conclusion:**

Our study shows a new pattern of reported depressive symptoms among first-and fifth-year medical students. In addition, the study suggests that depression is more common in female medical students as compared with their male counterparts. For future studies, we recommend using randomized sampling in a cohort study including all levels of medical students to further investigate and confirm the findings.

## Introduction

Depression, which is characterized by persistent sadness, is a highly prevalent and serious medical disease that affects more than 300 million individuals worldwide. It causes a social burden that involves the ill or depressed individual, his family, and community. In addition, depression burdens communities and countries economically and causes work-related issues, thus increasing suicide rates [1-3]. In the United States, around 10% of physician office visits are related to depression [4]. Approximately 43% of individuals with severe depressive symptoms reported hardships and serious obstacles at home and social events [5]. According to the National Vital Statistical Reports in 2014, there are 13.4 suicide deaths per 100,000 people [6]. Medical students show a higher prevalence of depressive symptoms than the general population [7]. For example, in a study of medical students in China, it was found that 51% of the sample had moderate to severe depression on the Patient Health Questionnaire-9 scoring system [8]. Another study comprising medical students in European and English-speaking countries shows variable prevalence of depression ranging from 6% to 66% [9]. In the United States, a prospective study was carried out to compare the risk of depression among medical students in their first and third years. The findings show a rise in the risk of depression in the third-year medical students compared with the risk in the first-year students [10]. Studies on Saudi Arabian medical students show high prevalence of depressive symptoms, comparable to that observed in other studies carried out in several countries [11]. To the best of the researchers’ knowledge, there are 6 studies concerned with the prevalence of depression in medical students in Saudi Arabia. High prevalence of depression was also observed in a cross-sectional study on pre-clinical years students. All in all, there was an increase in the risk of depression in third-year students in comparison with second-year students [12]. Our theory is that medical students are likely to experience depressive symptoms after prolonged exposure to the stressful environment of medical colleges, where they are expected to acquire a vast amount of knowledge in a restricted period of time. Our primary objective was to determine a pattern of reporting depressive symptoms among fifth-year and first-year medical students in Riyadh, Saudi Arabia. Our secondary objective was to compare the reporting of depressive symptoms between both genders.

## Materials and Methods

### Participants

The study sample comprises 470 students who are first-year and fifth-year students in Imam Muhammad Ibn Saud Islamic University and Princess Nourah Bint Abdul Rahman University in Riyadh, Saudi Arabia. This is a cross-sectional study that was carried out from October to November 2017.

### Inclusion and Exclusion Criteria

The inclusion criteria involved adult male and female medical students who agreed to participate in the study while attending the college of medicine in Imam Muhammad Ibn Saud Islamic University or Princess Nourah Bint Abdul Rahman University in Riyadh, Saudi Arabia. The exclusion criteria involved any medical students who did not give their consent or declined to cooperate.

### Data Collection

The researchers used the English version of Beck’s Depression Inventory (BDI). Previous studies were conducted with the English version of BDI and confirmed its validity and reliability as a tool to screen for and score the depressive symptoms [13]. The questionnaire includes 2 sections critical to our objectives. The first section consists of general demographic questions about age, gender, and academic year. The second section is the BDI questionnaire—English version. The sampling method was convenient, where our target population was first- and fifth-year medical students in Imam Muhammad Ibn Saud Islamic University and Princess Nourah Bint Abdul Rahman University in Riyadh.The original scoring system was used to interpret the data extracted using BDI. Beck’s questionnaire consisted of 21 questions with 4 choices to each question. Each choice equals a number of points varying from 0 to 3. The total lowest score in all the questions can be 0 points, and the highest possible score is 63 points. A high score means that the degree of depression the respondent has is severe. Finally, the system categorizes the scores into 6 levels: normal (1-10), mild mood disturbance (11-16), borderline clinical depression (17-20), moderate depression (21-30), severe depression (31-40), and extreme depression (>40).

### Ethical Statement

The students were approached during breaks or at the end of lectures. They were acquainted with the purposes of the study. Consents were taken, and full confidentiality of the participants and the obtained information was assured. The study received approval from the Office of Research Ethics at the Imam Muhammad Ibn Saud Islamic University, College of Medicine. (approval code: IRB-HAPO-01-R-011)

### Statistical Analysis

Statistical analysis was administered using the statistical package for social sciences (SPSS, Version 20.0. Armonk, NY: IBM Corp). Chi-square test was used as an analytical tool. P-value less than 0.05 was considered statistically significant.

## Results

The questionnaire was answered by 241 participants who met the inclusion criteria for the study. In Table-1, the age variable shows a bimodal distribution, where first-year students are aged 19±0.8 years, and fifth-year students are aged 23±2.1 years. Fifth-year students constitute 53.5% of the total sample, in which, male students accounted for 63.1% of the total sample (P=0.002). Table-2 demonstrates the difference between first-year and fifth-year male and female students. Regarding depression scale, male students scored higher than female students on the lower side of the scale in both years. Most of the first-year female students scored a higher level of depression compared with the male students (P=0.001). On the other hand, no difference in the levels of depression between males and females was observed in fifth-year students (P=0.4).

## Discussion

The purpose of this paper was to study and evaluate a pattern of reporting depression among medical school students belonging to different academic years. The researchers chose to apply the study to both male and female students from first and fifth year using the BDI scale.

### Difference Between First- and Fifth-Year Students

In comparison, first-year students scored a higher level of depression in both genders, which contradicts the theory proposed by this paper, which suggests that depression increases over time from the first level to the fifth. The proposed theory in this paper aligns with the findings of a previous study [8], which aimed to assess depressive symptoms by comparing the prevalence of depression among medical students in their first year and third year and showed a significantly increased risk of depression in third-year medical students compared with first-year medical students [8]. On the other hand, there was a Chinese study that compared the prevalence of depression among second- and third-year medical students and found that there was no significant difference among the students [9]. These contradictions between results could be due to societal differences. For example, in Saudi Arabia, most high schools do not put the students under heavy stress. When these students move to college and start their first year, they experience heavy stress because of the strict grading systems and demanding academic life. Our results regarding the difference in reporting of depressive symptoms among first- and fifth-year medical students could be attributed to the stress of college life because of the students’ inability to adjust to their new life, which imposes on them stress levels that they have not experienced before. For further research, the researchers recommend the inclusion of students from all the levels of medical college to clarify the pattern of association between reporting depressive symptoms and the academic levels of the students.

### Difference Between Males and Females

Our results show that males, in comparison to females, scored low in terms of the level of depression in both years, and this could be explained in the light of many factors such as biological and sociocultural factors. This confirms that prevalence of depression among females is higher than that in males, which is consistent with the data in the World Health Organization (WHO) booklet that states that depression prevalence in 2015 is 4.4% of the global population and is more common in females than in males. A study conducted in Saudi Arabia, Al-Qaseem University, investigating anxiety and depression in medical students, showed a high prevalence of depressive symptoms in females, which is consistent with other studies [12]. Multiple studies have demonstrated that females are at a greater risk of depression compared with males [14-17].To the best of the researchers’ knowledge, no studies were performed in Saudi Arabia to investigate the difference in experiencing depressive symptoms between preclinical year and clinical year medical students. Our limitations include the nature of the followed method, a cross-sectional study limited with convenient sampling, and the inclusion of only 2 medical colleges and 2 levels of medical students.

## Conclusion

To conclude, the study shows a new pattern of reported depressive symptoms among first- and fifth-year medical students, which evidently shows higher prevalence of symptoms being reported by first-year medical students compared to the fifth-year students. Moreover, the study is consistent with the factsheet of the WHO, which states that depression is more common in females than in males.

## Acknowledgment

The authors would like to profoundly thank Mr Ziad Almalki for his insightful comments and editing.

## Conflict of Interest

None declared.

**Table 1:** Demographic Data of the Respondents

**Variables**	**First year (n=112)**	**Fifth year (n=129)**	**Total**
**Age,y (mean±SD)**	19.5±0.890	23.67±2.18	21.7±2.6
**Gender, n (%)**			
Male	59 (38.8)	93 (61.2)	152 (63)
Female	53 (59.6)	36 (40.4)	89 (37)

**Table 2:** Depression Levels Among First- and Fifth-Year Medical Students

**Depression scale**	**Fifth year**		**First year**	
	Female	Male	Female	Male
**Normal**	12	46	9	32
**Mild mood disturbance**	8	14	12	12
**Borderline clinical depression**	5	7	8	8
**Moderate depression**	5	17	11	5
**Severe depression**	5	8	8	1
**Extreme depression**	1	1	5	1
**Total**	36	93	53	59
**P-value**	0.441		<0.001	
